# A Measuring System for Well Logging Attitude and a Method of Sensor Calibration

**DOI:** 10.3390/s140309256

**Published:** 2014-05-23

**Authors:** Yong Ren, Yangdong Wang, Mijian Wang, Sheng Wu, Biao Wei

**Affiliations:** 1 College of Communication Engineering, Chongqing University, Chongqing 400044, China; E-Mails: wydonglove@163.com (Y.W.); 20121202061@cqu.edu.cn (M.W.); 20121213065@cqu.edu.cn (S.W.); 2 College of Optoelectronic Engineering, Chongqing University, Chongqing 400044, China; E-Mail: weibiao@cqu.edu.cn

**Keywords:** attitude measurement, fluxgate, accelerometer, sensor calibration, tilt angle, azimuth angle

## Abstract

This paper proposes an approach for measuring the azimuth angle and tilt angle of underground drilling tools with a MEMS three-axis accelerometer and a three-axis fluxgate sensor. A mathematical model of well logging attitude angle is deduced based on combining space coordinate transformations and algebraic equations. In addition, a system implementation plan of the inclinometer is given in this paper, which features low cost, small volume and integration. Aiming at the sensor and assembly errors, this paper analyses the sources of errors, and establishes two mathematical models of errors and calculates related parameters to achieve sensor calibration. The results show that this scheme can obtain a stable and high precision azimuth angle and tilt angle of drilling tools, with the deviation of the former less than ±1.4° and the deviation of the latter less than ±0.1°.

## 1. Introduction

In oil, gas or geological exploration well logging work, acquiring the posture and orientation of the drilling tool in real-time [1,2], and transmitting the related information to the decision-making control side to adjust the action of drill accurately, will efficiently improve the quality of drilling engineering. Inertial navigation has an explicit definition of the space attitude information which will be reflected by the tilt angle, the azimuth angle and the tool face angle specifically [3]. Since the 1970s, inertial technology has developed rapidly and the corresponding tools, like dynamic debugging gyro inclinometers, optical fiber gyro inclinometers and so on have been implemented, but all these instruments have the shortcomings of high cost, large size and poor vibration resistance [4]. At present, the commonly used logging inclinometers include inclinometers based on fiber optic gyro or magnetic sensors. The former, free from any external magnetic disturbance, is of large volume, and high cost with accumulative errors existing in the measurement while drilling; the latter, with small volume and low cost, acts instantly when measuring and has no accumulated error [5,6].However, the existing logging inclinometers based on magnetic sensor systems still have some disadvantages such as low systematic digitalization, low precision of the attitude results and incomprehensive error calibration or complex calibration methods. In the existing products used in the oil drilling industry, the general azimuth measuring accuracy is ±3° and the tilt angle one is ±0.3°.

Consequently this paper proposes a scheme to develop a measuring system for well logging attitude by using a MEMS three-axis accelerometer and a three-axis fluxgate sensor which has small size [7], light weight, low power consumption and no gyroscope. As there are various sensor errors which affect the accuracy of the calculated angle [8–11], it is particularly important to find a way to effectively reduce these errors. Traditionally, the inertial navigation sensor calibration algorithms described in many literatures includes the commonly used ferromagnetic effect calibration method as well as genetic algorithm and BP neural network methods and the least square method. The ferromagnetic effect algorithm is used to obtain the maximum and minimum output by rotating the magnetic sensor and thereby acquire the offset and scale factor of the magnetic sensor. This method with its simple principle is easy to operate and implement, but on the other hand, it only completes the calibrations of the hard iron interference errors and tri-axial sensitiveness errors and fails to realize the calibration of alignment errors which is an indispensible process to achieve the accord between two sensor axes and equipment axes. To obtain calibration parameters with a genetic algorithm or BP neural network in practice, which is theoretically feasible, involves a large amount of programming with computational complexity, so it is generally not used. In addition, many of the traditional navigation algorithms are not entirely suitable for well logging. On the basis of the traditional calibration method [12–15], a newly proposed calibration method can improve the accuracy.

## 2. Theory of Measurement

### 2.1. Systematic Design

**Figure 1.**
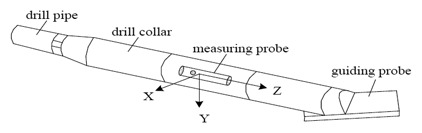
The configuration of a logging tool.

For directional well logging attitude measurement, the guiding parts mainly include a sensor module, signal acquisition module, microcontroller module and communication interface (SPI, SCI). All these component are assembled in a probe casing with small size and diameter (φ35 mm × 260 mm), and a PC works as the remote control and display device. The mechanical configuration of a logging tool can be expressed as shown in Figure 1. It includes a variety of mechanical fittings and a guiding probe which is the core measurement device.

**Figure 2.**
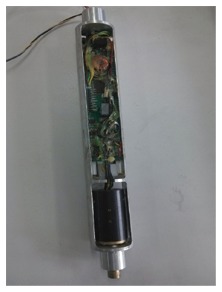
The measuring probe.

A picture of the designed measuring probe is shown in Figure 2. The measuring probe is used to measure and respond in real time to the posture information of the drilling tool, and it is also designed as an aluminum cylinder structure, so that the device is compact, nonmagnetic and especially convenient for other mechanical adaptations and subsequent turntable experiments.

**Figure 3.**
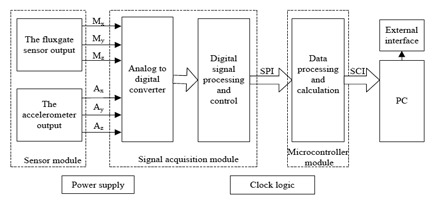
The system principle block diagram.

The main function of the measuring probe based on accelerometers and fluxgate sensors designed in this paper is to measure the azimuth angle and tilt angle of the well logging tool. The system principle block diagram of the hardware for the measuring device is shown in Figure 3. The accelerometer uses an ADIS16210 which combines a high accuracy MEMS tri-axial acceleration sensor with ±1.7 g measurement range and ±0.061 mg sensitivity. The selected tri-axial fluxgate sensor measurement range is 0∼±100,000 nT and the resolution can reach 1 nT. The main microcontroller uses a MC9S12XEP100 Freescale MCU.

### 2.2. Mathematical Calculating of Attitude Angle

The equipment used for measuring the attitude of a directional well logging tool is usually called a well logging inclinometer. Acquiring the posture and orientation uses the gravity field and magnetic field which have relative stability characteristics. Under different orientations, the fluxgate sensor and acceleration sensor data output will have different values. By 3D coordinate rotation and transformation, the current attitude angle and azimuth angle of the equipment can be uniquely determined.

**Figure 4.**
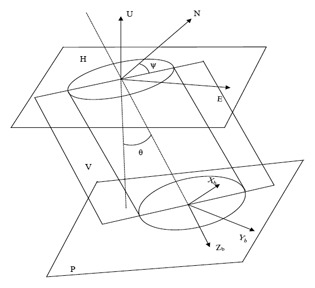
Attitude angle diagram.

As shown in Figure 4, the navigation coordinates E/N/U are defined as east/north/up based on the right-hand rule, and the device body coordinates *X_b_/Y_b_/Z_b_* are defined as forward/right/down based on the right-hand rule. H represents the horizontal plane; V the borehole bending plane; and P the drill cross section. The tilt angle (*θ*) is an angle between *Z_b_* axis and the vertical direction, and the azimuth angle (*Ψ*) is an angle between the horizontal projection of *Z_b_* axis and north.

**Figure 5.**
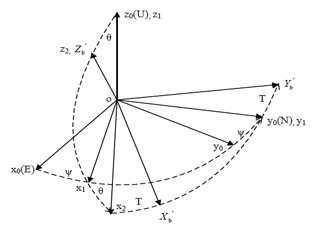
The process of coordinate transformation.

A device can always transform a fixed location to the current location through a rotation matrix. As shown in Figure 5, the navigation coordinates o*x_0_y_0_z_0_* (oENU) are used as a reference frame and clockwise rotation is positive. Firstly rotate an angle of *ψ* around oz_0_ to the coordinates o*x_1_y_1_z_1_*, then an angle of *θ* around o*y_1_* to the coordinates o*x_2_y_2_z_2_*, finally an angle of *T* around o*z_2_* to the coordinates o*X′_b_Y′_b_Z′*_b_ which are the device body coordinates.

Therefore the rotation matrix can be expressed as Equation (1) in which *ψ* is the azimuth angle, *θ* is the tilt angle, and *T* is the tool face angle. The relationship between *X′_b_Y′_b_Z′_b_* and *x_0_y_0_z_0_* is expressed by Equation (2):

(1) $$C=R_{T}R_{\theta }R_{\psi }=\left[\begin{array}{ccc} \cos\psi \cos\theta \cos T-\sin\psi \sin T & \sin\psi \cos\theta \cos T+\cos\psi \sin T & -\sin\theta \cos T\\ -\cos\psi \cos\theta \sin T-\sin\psi \cos T & -\sin\psi \cos\theta \sin T+\cos\psi \cos T & \sin\theta \sin T\\ \cos\psi \sin\theta & \sin\psi \sin\theta & \cos\theta \end{array}\right]$$

(2) $$\left[\begin{array}{c} {X_{b}}^{'}\\ {Y_{b}}^{'}\\ {Z_{b}}^{'} \end{array}\right]==C•\left[\begin{array}{c} X_{0}\\ Y_{0}\\ Z_{0} \end{array}\right]=\left[\begin{array}{ccc} \text{cos}\psi \text{cos}\theta \text{cos}T-\text{sin}\psi \text{sin}T & \text{sin}\psi \text{cos}\theta \text{cos}T+\text{cos}\psi \text{sin}T & -\text{sin}\theta \text{cos}T\\ -\text{cos}\psi \text{cos}\theta \text{sin}T-\text{sin}\psi \text{cos}T & -\text{sin}\psi \text{cos}\theta \text{sin}T+\text{cos}\psi \text{cos}T & \text{sin}\theta \text{sin}T\\ \text{cos}\psi \text{sin}\theta & \text{sin}\psi \text{sin}\theta & \text{cos}\theta \end{array}\right]•[\begin{array}{c} x_{0}\\ y_{0}\\ z_{0} \end{array}$$

Let *A_x_*, *A_y_* and *A_z_* be the normalized accelerometer output values after filtering and *M_x_*, *M_y_* and *M_z_* the normalized fluxgate sensor output values after filtering. In the local horizontal plane, *A_x_*_0_ = *A_y_*_0_ = 0, *A_z_*_0_ = +1 *g*, then Equation (2) becomes:

(3) $$\left[\begin{array}{c} A_{x}\\ A_{y}\\ A_{z} \end{array}\right]=C•\left[\begin{array}{c} 0\\ 0\\ 1 \end{array}\right]$$

Therefore, *θ* and *T* can be calculated as follows:

(4) $$\mathrm{dev}=I=\arctan\frac{\sqrt{{A^{2}}_{x}+{A^{2}}_{y}}}{A_{z}}$$

(5) $$T=-\arctan(A_{y}/A_{x})$$

In the local horizontal plane, the values of the fluxgate sensor in the E/N/U direction can be calculated as *M_x_*_0_ = 0, *M_y_*_0_ = *M*cos*φ*, *M_z_*_0_ = *M*sin*φ*. Here *M* is the magnetic value of the local geomagnetic field and *φ* is the local latitude, so Equation (2) becomes:

(6) $$\left[\begin{array}{c} M_{x}\\ M_{y}\\ M_{z} \end{array}\right]=C•\left[\begin{array}{c} 0\\ M\cos\phi \\ M\sin\phi \end{array}\right]$$

And *ψ* can be calculated as follows:

(7) $$\text{d}az=\psi =\arctan\frac{M_{z}-A_{z}Q}{M_{x}A_{y}-M_{y}A_{x}}$$

Here *Q* = *M_x_* × *A_x_* + *M_y_* × *A_y_* + *M_z_* × *A*_z_. Equation (7) shows that the value of *daz* is not related to *φ* or *M*.

## 3. The Proposed Calibration Method

The acceleration sensors and fluxgate sensors used in this paper have been strictly calibrated before they leave the factory, and their accuracy has a certain guarantee. However, in considering the overall measurement equipment, after the device is assembled in the mechanical aspects, this will cause new errors due to the inevitable mechanical installation axial misalignment, circuit effects, hard-iron interference, *etc*.

The actual device axis (Z-axis) is defined as the reference axis for calibration. Taking the errors of the sensors and the types of errors after completion of the sensors assembly into unified consideration and fusion processing, the errors of the system after assembly are mainly the result of four aspects: (1) Misalignment error is defined as the angles between the sensor sensing axes and the device body axes, caused by manufacturing and installation; (2) Hard-iron interference magnetic field is normally generated by ferromagnetic materials with permanent magnetic fields that are part of the device structure. These materials could be permanent magnets, magnetized iron or steel; (3) A soft-iron interference magnetic field is generated by the uncertain magnetically soft materials surrounding the device or the items inside current carrying traces on the PCB. For some platforms, hard-iron interference is the primary source of error and soft-iron distortion is minimal or non-existent; (4) The scale factor error is defined as the mismatch of the sensitivity of the sensor sensing axes. Ideally, the three-axis sensors that make up the triad are identical. In reality, however, this may not be the case. Each sensor channel may have different sensitivities. Calibration is designed to reduce these errors.

To calibrate these errors, the existing least square method is, through the establishment of multi-parameter equation, used to collect multiple samples to calculate the calibration parameters. However, they have the following shortcomings. First, some perform the error correction incompletely. For example, it only corrects two or three of the four errors. Second, the number of samples limits the accuracy of the parameters. Third, a variety of established equations are not simple and clear with complicated solving processes for the parameter equations. Additionally, the ellipsoidal model is also established in some papers to achieve the magnetic calibration, but it involves a complex parameter solving process and adopts a simplified approximation to replace the parameter values, which cannot fully represent the types of errors. This paper establishes a comprehensive error model based on the above four errors, and uses the least square method to calculate a calibration matrix. A simple and practical calibration process is thus designed.

### 3.1. Accelerometer Calibration Model

The error model of the accelerometer can be expressed as follows [16,17]:

(8) $$\left[\begin{array}{c} A_{x}\\ A_{y}\\ A_{z} \end{array}\right]={\left[A_{m}\right]}_{3\times 3}\cdot \left[\begin{array}{ccc} \frac{1}{k_{x}} & 0 & 0\\ 0 & \frac{1}{k_{y}} & 0\\ 0 & 0 & \frac{1}{k_{z}} \end{array}\right]\cdot \left[\begin{array}{ll} A_{x} & -e_{x}\\ A_{y} & -e_{y}\\ A_{z} & -e_{z} \end{array}\right]=\left[\begin{array}{ccc} a_{11} & a_{12} & a_{13}\\ a_{21} & a_{22} & a_{23}\\ a_{31} & a_{32} & a_{33} \end{array}\right]\cdot \left[\begin{array}{c} A_{x0}\\ A_{y0}\\ A_{z0} \end{array}\right]+\left[\begin{array}{c} a_{10}\\ a_{20}\\ a_{30} \end{array}\right]$$

Here [*A_m_*]_3×3_ is a 3 × 3 misalignment matrix between the accelerometer sensing axes and the device body axes; *k*_i_(*i* = *x,y,z*) is the scale factor and *e*_i_(*i* = *x,y,z*) is the offset, *a*_10_∼*a*_33_ are the calibration parameters, *A_x_*_0_, *A_y_*_0_, *A_z_*_0_ are raw measurements and *A_x_*, *A_y_*, *A_z_* are normalized values. Equation (8) can then be rewritten as:

(9) $${\left[\begin{array}{ccc} A_{x} & A_{y} & A_{z} \end{array}\right]}_{n\times 3}={\left[\begin{array}{llll} A_{x0} & A_{y0} & A_{z0} & 1 \end{array}\right]}_{n\times 4}\cdot \left[\begin{array}{lll} a_{11} & a_{21} & a_{31}\\ a_{12} & a_{22} & a_{32}\\ a_{13} & a_{23} & a_{33}\\ a_{14} & a_{20} & a_{30} \end{array}\right]$$

or ***N*** = **A·*a*** where, Matrix ***a*** is composed of 12 calibration parameters that need to be determined. Matrix ***A*** is composed of sensor raw data collected at several stationary positions. Matrix ***N*** is the known normalized Earth gravity vector. The goal of the accelerometer calibration is to determine 12 parameters from *a*_10_ to *a*_33_, and with any given normalized values in a position, the raw measurements can be obtained. For example, at *Z_b_* down position where the tilt angle scale indicating on the standard turntable is zero, [*A_x_ A_y_ A_z_*] = [0 0 1] and a set of accelerometer raw data *A_x_*_0_, *A_y_*_0_ and *A_z_*_0_ can be collected. According to the standard turntable, we choose 10 positions with *X_b_* down and up, *Y_b_* down and up *Z_b_* down and up, *A_x_* = 0, *A_y_* = ±0.707 *g*, *A_z_* = −0.707 *g* and *A_x_* = ±0.707 *g*, *A_y_* = 0, *A_z_* = −0.707 g and collect several a second set of accelerometer raw data at each position with known *A_x_*_0_, *A_y_*_0_ and *A_z_*_0_. The calibration parameter matrix ***a*** can be determined by the least square method as:

(10) $$a={\left[A^{T}\cdot A\right]}^{-1}\cdot A^{T}\cdot N$$

If the raw data of accelerometer is [*A_x_*_1_ *A_y_*_1_ *A_z_*_1_], the calibrated data which be used to calculate the attitude angle can be expressed as [*A_x_*_2_ *A_y_*_2_ *A_z_*_2_] = [*A_x_*_1_ *A_y_*_1_ *A_z_*_1_ 1]·***a***.

**Figure 6.**
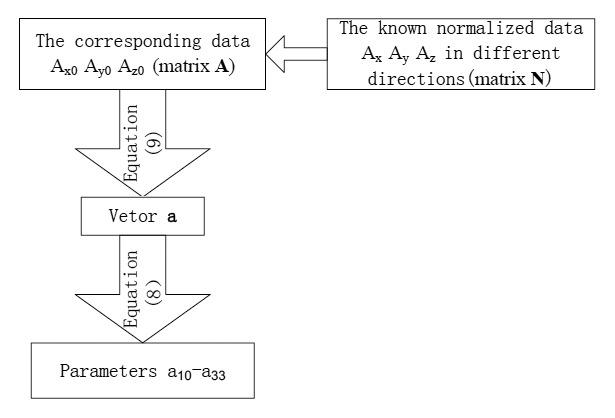
The calculation process of accelerometer calibration parameter.

The calculation process of the accelerometer calibration parameters is shown in Figure 6.

### 3.2. Fluxgate Sensor Calibration Model

The relationship between the normalized data *M_x_*, *M_y_*, *M_z_* and the magnetic sensor raw measurements *M*_x0_, *M*_y0_, *M*_z0_ can be expressed as Equation (11) [18]:

(11) $$\left[\begin{array}{c} M_{x}\\ M_{y}\\ M_{z} \end{array}\right]={\left[M_{m}\right]}_{3\times 3}\cdot \left[\begin{array}{ccc} \frac{1}{k_{mx}} & 0 & 0\\ 0 & \frac{1}{k_{my}} & 0\\ 0 & 0 & \frac{1}{k_{mz}} \end{array}\right]\cdot \left[\begin{array}{ll} M_{x0} & -e_{mx}\\ M_{y0} & -e_{my}\\ M_{z0} & -e_{mz} \end{array}\right]=\left[\begin{array}{ccc} m_{11} & m_{12} & m_{13}\\ m_{21} & m_{22} & m_{23}\\ m_{31} & m_{32} & m_{33} \end{array}\right]\cdot \left[\begin{array}{ll} M_{x0} & -m_{10}\\ M_{y0} & -m_{20}\\ M_{z0} & -m_{30} \end{array}\right]$$

Here [*M_m_*] is a 3 × 3 misalignment matrix between the magnetic sensor sensing axes and the device body axes; *k_m_*_i_(*i* = *x,y,z*) is the scale factor and *e_m_*_i_(*i* = *x,y,z*) is the offset caused by hard-iron distortion; [*M*_s_] is a 3 × 3 matrix caused by soft-iron distortion. The goal of the magnetic sensor calibration is to determine the parameters from *m_10_* to *m_33_*, and with any given raw measurements at arbitrary positions, the normalized values can be obtained. It is always good to know if the device has the above interference before choosing which model to use for the identification of the calibration parameters, tilted ellipsoid, or non-tilted ellipsoid. This can be done by performing 3D rotations in a clean environmental area. Then we plot the collected magnetic sensor raw data with MATLAB to check if there is any interference field inside the device. This set of data is not used for the subsequent magnetic sensor calibration. However in practical situations, three 2D full round rotations may not be easy to perform. Then an amount of 3D rotation data can be used for rough field calibration. If there is soft-iron distortion, the 3D rotations show a tilt ellipsoid which can be described by the following equation:

(12) $$\frac{{\left(x-x_{0}\right)}^{2}}{a^{2}}+\frac{{\left(y-y_{0}\right)}^{2}}{b^{2}}+\frac{{\left(z-z_{0}\right)}^{2}}{c^{2}}+\frac{\left(x-x_{0})(y-y_{0}\right)}{d^{2}}+\frac{\left(x-x_{0})(z-z_{0}\right)}{e^{2}}+\frac{\left(y-y_{0})(z-z_{0}\right)}{f^{2}}=R^{2}$$

Here *x*_0_, *y*_0_, *z*_0_ are the offsets *e_m_*_i_(*i = x, y, z*), *x, y, z* are magnetic sensor raw data, *a*, *b*, *c*, are the semi-axes lengths, *d*, *e*, *f*, are cross axis effect to make the ellipsoid tilted, *R* is a constant of the Earth's magnetic field strength. Actually, the designed device doesn't utilize any magnetically soft materials in the hardware design and mechanical assembles, and there are no soft materials in the application environment. The calibration parameters are acquired in the open field which has no magnetically soft materials. Additionally, the soft-iron interference magnetic field from the current on PCB is weak and fixed and it is calibrated together with the hard-iron interference. It is discovered that the model is a normal ellipsoidal without tilt described in Figure 9a when MATLAB is used to simulate and test the magnetic data of instruments, so the soft-iron interference is negligible and can be ignored. The ellipsoid can be simplified as the following equation:

(13) $$\frac{{\left(x-x_{0}\right)}^{2}}{a^{2}}+\frac{{\left(y-y_{0}\right)}^{2}}{b^{2}}+\frac{{\left(z-z_{0}\right)}^{2}}{c^{2}}=R^{2}$$

Here *x*_0_,*y*_0_,*z*_0_ are the offsets *e_m_*_i_(*i = x, y, z*) caused by hard-iron distortion. *x,y,z* are magnetic sensor raw data *M_x_*, *M_y_* and *M_z_ a*, *b*, *c* are the semi-axes lengths, *R* is a constant of the Earth's magnetic field strength. Equation (13) can be rewritten as:

(14) $$x^{2}=[\begin{array}{llllll} x & y & z & -y^{2} & -z^{2} & 1 \end{array}]\cdot \left[\begin{array}{c} 2x_{0}\\ \frac{a^{2}}{b^{2}}2y_{0}\\ \frac{a^{2}}{c^{2}}2z_{0}\\ \frac{a^{2}}{b^{2}}\\ \frac{a^{2}}{c^{2}}\\ aR^{2}-{x_{0}}^{2}-\frac{a^{2}}{b^{2}}y_{0}^{2}-\frac{a^{2}}{c^{2}}z_{0}^{2} \end{array}\right]$$

Then:

(15) $${\text{X}}_{n\times 1}={\text{M}}_{n\times 6}\cdot {\text{I}}_{6\times 1}$$

The least square method can be applied to determine the parameters ***I*** vector as:

(16) $$\text{I}={\left[{\text{M}}^{T}\text{M}\right]}^{-1}{\text{M}}^{T}\cdot \text{X}$$

Then:

(17) $$\{\begin{array}{c} e_{mx}=x_{0}=\frac{\text{I}(1)}{2}\\ e_{my}=y_{0}=\frac{\text{I}(2)}{2\cdot \text{I}(4)}\\ e_{mz}=z_{0}=\frac{\text{I}(3)}{2\cdot \text{I}(5)} \end{array}$$

and:

(18) $$\{\begin{array}{c} A=a^{2}R^{2}=\text{I}(6)+{x_{0}}^{2}+\text{I}(4)\cdot {y_{0}}^{2}+\text{I}(5)\cdot {z_{0}}^{2}\\ B=b^{2}R^{2}=A/\text{I}(4)\\ B=c^{2}R^{2}=A/\text{I}(5) \end{array}$$

Let:

(19) $$\begin{array}{ccc} x_{1}=M_{x0}-e_{mx}, & y_{1}=M_{y0}-e_{my}, & z_{1}=M_{z0}-e_{mz} \end{array}$$

Then Equation (13) becomes:

(20) $$\frac{x_{1}^{2}}{A}+\frac{y_{1}^{2}}{B}+\frac{z_{1}^{2}}{C}=1$$

Therefore:

(21) $$\{\begin{array}{c} k_{mx}=\sqrt{A}\\ k_{my}=\sqrt{B}\\ k_{mz}=\sqrt{C} \end{array}$$

Let:

(22) $$\begin{array}{ccc} x_{2}=x_{1}/k_{mx}, & y_{2}=y_{1}/k_{my}, & z_{2}=z_{1}/k_{mz} \end{array}$$

and:

(23) $$x_{2}^{2}+y_{2}^{2}+z_{2}^{2}=1$$

Up to now, *k_m_*_i_(*i* = *x, y, z*) the scale factor, *e_m_*_i_(*i = x, y, z*) the offset caused by hard-iron distortion, and the [*M*_s_]_3×3_ matrix caused by soft-iron distortion have been determined.

Let ***M****_m_*_×3_ = [*x*_2z_ *y*_2z_ *z*_2z_] be the Z_b_ down rotation circle data after scale factor, hard-iron and soft-iron correction:

(24) $${\text{X}}_{m\times 1}=\sqrt{x_{2z}^{2}+y_{2z}^{2}+z_{2z}^{2}}$$

Then:

(25) $${\text{I}}_{3\times 1}={\left[{\text{M}}^{T}\text{M}\right]}^{-1}{\text{M}}^{T}\cdot \text{X}$$

So the normalized rotation vector for Z_b_ down rotation is:

(26) $${\text{w}}_{z}=\text{I}/\sqrt{\text{I}{\left(1\right)}^{2}+\text{I}{\left(2\right)}^{2}+\text{I}{\left(3\right)}^{2}}$$

Similarly, the normalized rotation vectors ***w****_x_* and ***w****_y_* for *X_b_* down rotation and *Y_b_* down rotation can be determined. Then the final misalignment compensation matrix is:

(27) $${\left[{\text{M}}_{m}\right]}_{3\times 3}=\left[\begin{array}{ccc} {\text{w}}_{x} & {\text{w}}_{y} & {\text{w}}_{z} \end{array}\right]$$

**Figure 7.**
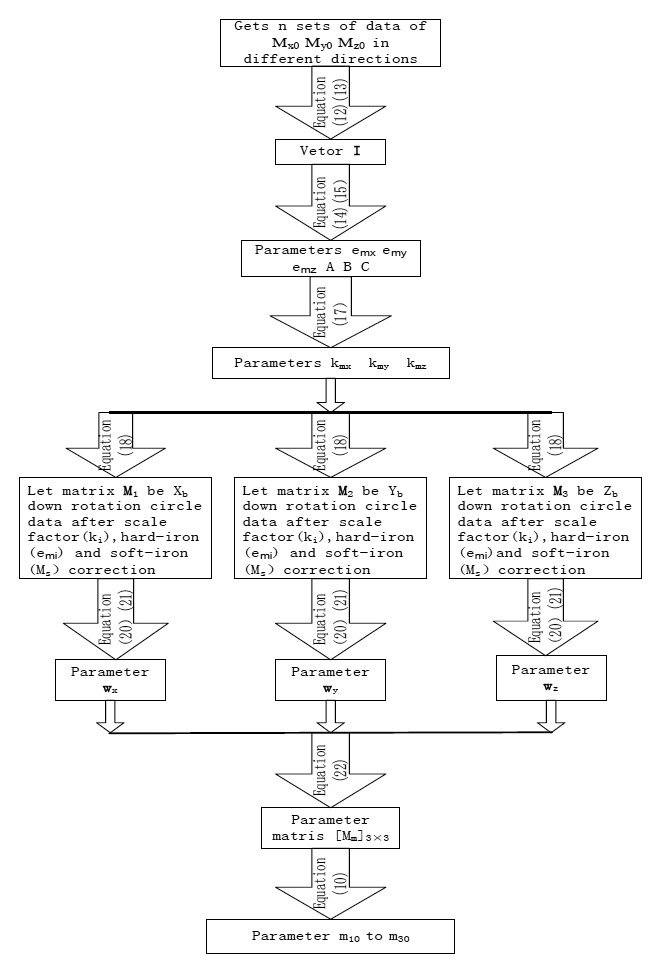
The calculation process of fluxgate sensor calibration parameters.

So the parameters from *m*_10_ to *m*_33_ can be calculated by Equation (11). The calculation process of fluxgate sensor calibration parameter is shown in Figure 7.

## 4. Experiments and Analysis

For attitude measurement of exploring casinga in production and practice, a non-magnetic and omnibearing standard turntable, which can display and inspect the tilt angle (ranging 0 to ±90°) and the azimuth (ranging 0 to 360°), is often used as the test platform. Moreover the standard turntable is strictly adjusted by precise third party calibration instruments before the experiment, and then we can examine the resulting precision of the angle measurement based on the turntable. The adjusted turntable can guarantee the tilt angle is 0° and the azimuth is 0° when it is at the zero position, and the reading error of the turntable calibration is within ±0.1°; That is, the experimental turntable guarantees the tile angle scale indicates 0° with the exploring casing is vertically direct to the ground, and it also guarantees the azimuth scale indicates 0° with the exploring casing is directed to the magnetic north. The system adopts the output value of the final calculated measurement result by comparing the current value of the turntable calibration as the relative error for measurements, which is a conventional method for cylindrical, probe tubular underground inclinometer devices, this method is simple, easily used, and also able to test the measurement precision.

**Figure 8.**
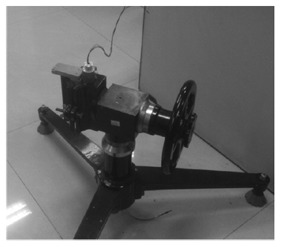
The calibration and experiment platform.

The test calibration and experiment platform is shown in Figure 8. The 3D movable platform is used to collect multiple sets of data by soft filtering. The more data are collected, the more accurate the calibration parameters that will be adopted in the least square method to calculate the 12 calibration parameters of the accelerometer and the fluxgate sensor separately. In the experiment, the calibration parameters of the fluxgate sensor are calculated by reading 360 datum of the fluxgate sensor under different spatial orientations.

**Figure 9.**
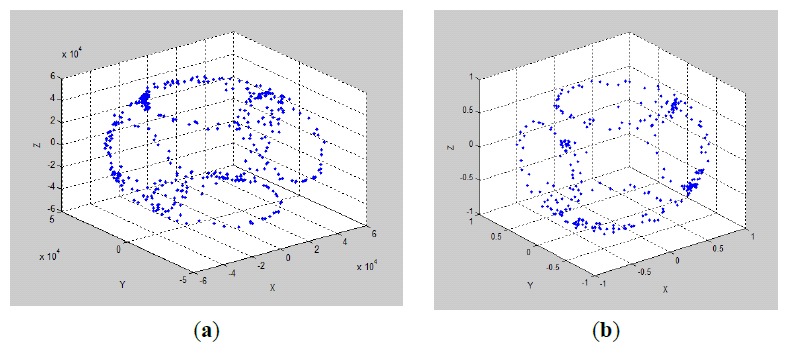
Comparision of fluxgate output data before and after calibration.

There is a standard method to show the fluxgate calibration results. As shown in Figure 9, Figure 9a is an ellipsoid before fluxgate calibration and Figure 9b is a normal sphere, which proves that the fluxgate errors of the scale factor and center offset have been calibrated.

The turntable is use to test the designed inclinometer. The inclinometer needs keep the same center with the turntable. Taking eight tilt angles (3°, 15°, 30°, 60°, −3°, −15°, −30°, −60°), and rotating eight azimuth angles, respectively (0°, 45°, 90°, 135°, 180°, 225°, 270°, 315°) at each tilt angle, we then record and save the current measurement result values. Comparing these values with the standard tilt angle and azimuth angle, Table 1 shows that *dev* is the tilt angle calculated by this scheme, *dev*_0_ is the tilt angle calculated with no accelerometer calibration, *v_err*_0_ is the deviation between *dev*_0_ and the stander value of tilt and *v_err* is the deviation between *dev* and the standard tilt value.

Table 2 shows that *daz* is the azimuth angle calculated by this scheme, *daz*_0_ is the azimuth angle calculated after fluxgate sensor calibration using the traditional ellipse matching error compensation algorithm [12,13], *z_err*0 is the deviation between *daz*_0_ and the stander values of azimuth and *z_err* is the deviation between *daz* and the standard value of azimuth. *dev*_0_, *dev*, *daz*_0_ and *daz* are angles which have the biggest deviation compared with the standard values when recorded.

The two forms of data above show that the azimuth angle error with traditional compensation will reach ±4° and the tilt angle error with no calibration will reach ±0.4°. As for the errors of the accelerometer (scale factor error, misalignment error, external disturbance) and fluxgate sensor (hard-iron interference, soft-iron interference, scale factor error, misalignment error), the azimuth error is less than ±1.4° and tilt angle error is less than ±0.1° after calibration by the proposed method, so we can state that the calibration method improves the accuracy of the attitude angle and is proved to be effective, so it can be applied to actual well logging work.

## 5. Conclusions

Based on the design of a measuring system for well logging attitude, this paper proposes a concise formula for attitude angle calculation, and establishes mathematical models to calibrate errors. Taking the error of the sensor itself and the four main types of errors after completion of the sensor assembly into unified consideration and fusion processing, a comprehensive error model has been established. Based on this model, a simple and practical calibration process is designed, which can be completed using a standard test turntable. Furthermore, it can complete the calibration of equipment errors, including installation errors and sensor errors, so the measurement accuracy can then be improved. The test results show that these schemes are effective and highly precise. The designed measuring equipment has utility in engineering applications and has the characteristics of small size, high integration, low-cost and easy adaptation to other devices. The measuring deviation of azimuth angle and tilt angle of drilling tools are thus greatly reduced. The calibration parameters can be calculated in advance and be used in the soft system to acquire the attitude angle of directional well logging.

## Figures and Tables

**Table 1.** The tilt angle measurement data.

**Standard Values**	***dev*_0_**	***z_err*0**	***dev***	***v_err***
3°	2.76°	−0.24°	2.91°	−0.09
15°	14.73°	−0.27°	14.93°	−0.07
30°	29.71°	−0.29°	29.98°	−0.02
60°	59.62°	−0.38°	59.95°	−0.05
−3°	−3.28°	−0.28°	−3.03°	−0.03
−15°	−15.33°	−0.33°	−15.06°	−0.06
−30°	−30.37°	−0.37°	−30.08°	−0.08
−60°	−60.40°	−0.40°	−60.03°	−0.03

**Table 2.** The azimuth angle measurement data.

**Standard Values**	***daz*_0_**	***z_err*0**	***daz***	***z_err***
0°	0.85°	0.85°	0.57°	0.57°
45°	41.73°	−3.27°	45.14°	0.14°
90°	87.89°	−2.12°	91.35°	1.35°
135°	131.45°	−3.55°	136.19°	1.19°
180°	184.11°	4.11°	181.36°	1.36°
225°	228.05°	3.05°	223.98°	−1.02°
270°	273.4°	3.4°	269.12°	−0.88°
315°	318.20°	3.2°	313.81°	−1.19°
